# Identification of Splice Variants and Isoforms in Transcriptomics and Proteomics

**DOI:** 10.1146/annurev-biodatasci-020722-044021

**Published:** 2023-08-10

**Authors:** Taojunfeng Su, Michael A.R. Hollas, Ryan T. Fellers, Neil L. Kelleher

**Affiliations:** 1 Department of Molecular Biosciences, Northwestern University, Evanston, Illinois, USA;; 2 Proteomics Center of Excellence, Northwestern University, Evanston, Illinois, USA; 3 Department of Chemistry, Northwestern University, Evanston, Illinois, USA

**Keywords:** alternative splicing, short-read RNA-seq, long-read RNA-seq, top-down mass spectrometry, protein isoform, proteoform

## Abstract

Alternative splicing is pivotal to the regulation of gene expression and protein diversity in eukaryotic cells. The detection of alternative splicing events requires specific omics technologies. Although short-read RNA sequencing has successfully supported a plethora of investigations on alternative splicing, the emerging technologies of long-read RNA sequencing and top-down mass spectrometry open new opportunities to identify alternative splicing and protein isoforms with less ambiguity. Here, we summarize improvements in short-read RNA sequencing for alternative splicing analysis, including percent splicing index estimation and differential analysis. We also review the computational methods used in top-down proteomics analysis regarding proteoform identification, including the construction of databases of protein isoforms and statistical analyses of search results. While many improvements in sequencing and computational methods will result from emerging technologies, there should be future endeavors to increase the effectiveness, integration, and proteome coverage of alternative splicing events.

## 1. INTRODUCTION

**Figure 1:**
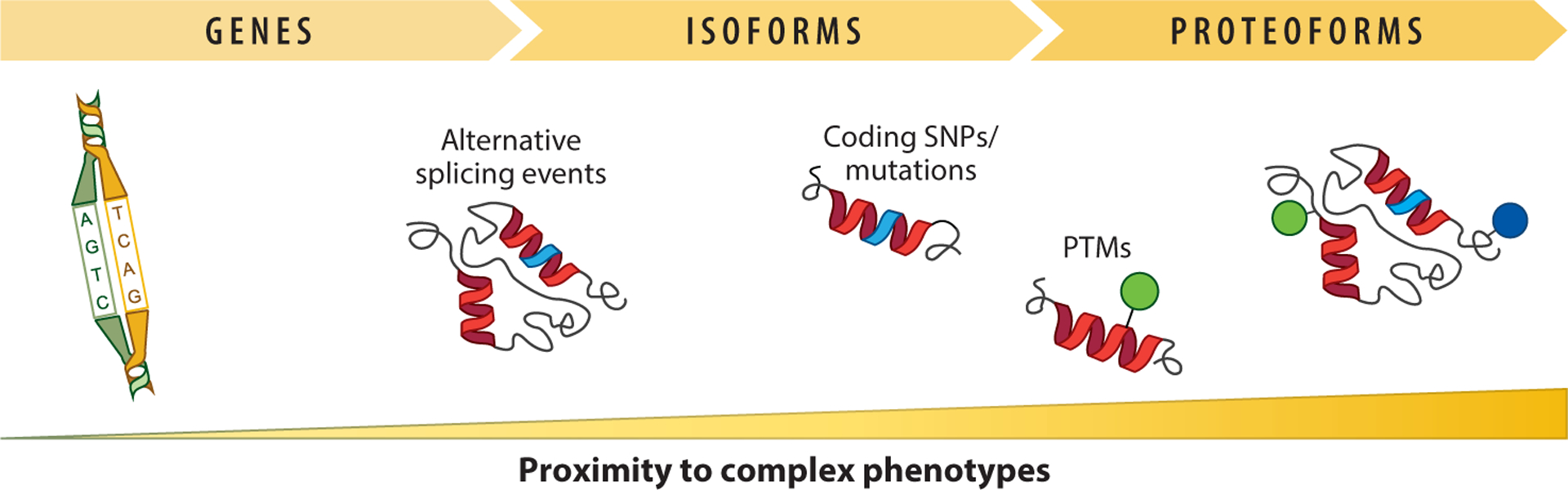
From genes to proteoforms. This version of the central dogma highlights the sources of variation at the DNA, RNA, and protein level that create proteoforms (unique protein molecules). The direct measurement of proteoforms thus captures all sources of protein variation, including allele expression, isoforms, and combinations of PTMs. As such, proteoforms correlate more tightly to complex phenotypes in the human population relative to other biomolecules but are challenging to measure. Abbreviations: PTM, posttranslational modification; SNP, single-nucleotide polymorphism.

Alternative splicing is the process of selecting different combinations of exons, the segments of a gene that code for a particular region of protein sequence, within a messenger RNA (mRNA) precursor to produce variably spliced mRNAs (1). The spliced mRNA generated from a gene will serve as a template for protein translation, generating proteins with different sequences (i.e., protein isoforms) in humans (2). Protein isoforms may carry mutated amino acids derived from coding single-nucleotide polymorphisms (cSNPs) and may also be posttranslationally modified in different biological contexts (Figure 1). As a result, protein isoforms, cSNPs, and posttranslational modifications (PTMs) are three elements that define a proteoform, a particular form of a protein that contributes to phenotypic traits in organisms (3). Previous investigations have found a variety of human diseases associated with alternative splicing events. For example, skipping of the eleventh exon of the *BRCA1* gene has been implicated in early-onset breast cancer (4), and elevated expression of the p53 isoform with retention of the ninth intron has been associated with reduced survival in patients with uterine serous carcinoma (5). Therefore, knowing which isoform is expressed in human diseases can have a huge benefit for drug development and screening assays (6, 7), disease biomarker discovery (8), and disease mechanism studies (9–11).

**Figure 2:**
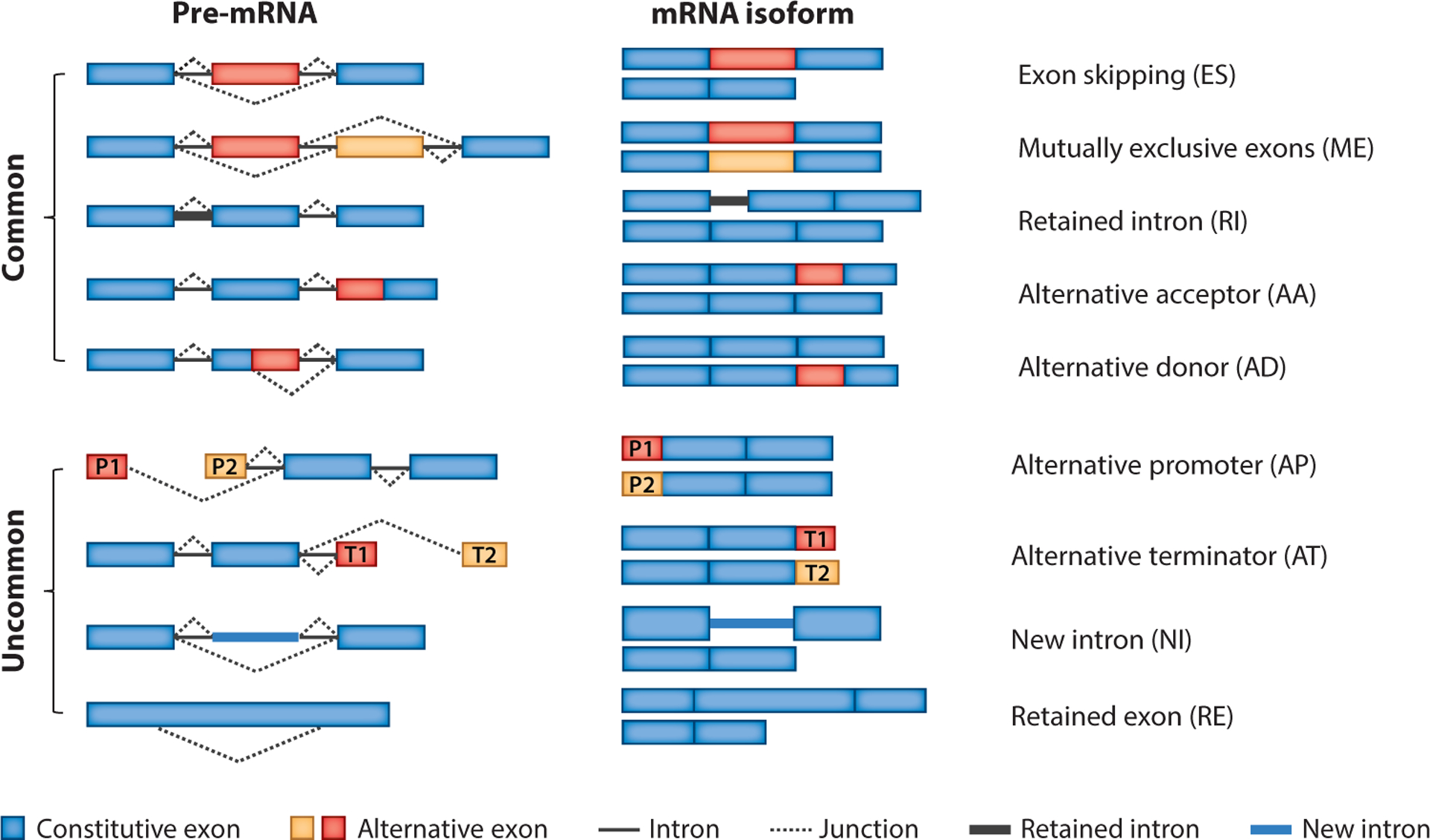
Illustration of the nine types of alternative splicing in the present review, including exon skipping, mutually exclusive exons, retained intron, alternative acceptor, alternative donor, alternative promoter, alternative terminator, new intron, and retained exon. P1 and P2 represent two possible promoters and T1 and T2 represent two terminators. Abbreviations: mRNA, messenger RNA; pre-, precursor.

Alternative splicing can produce a plethora of splice variants, which are typically categorized into five major groups (Figure 2), defined as exon skipping, alternative donor, alternative acceptor, mutually exclusive exon, and retained intron (1, 12). With the utilization of modern sequencing techniques, which enable both high sequencing depth and high coverage, several other alternative splicing events have been identified. New intron and retained exon were first identified in two bean plants, *Phaseolus vulgaris* and *Glycine max* (13). New intron is defined as a splicing site that appears in a reported exon, and a retained exon is defined as a new exon that replaces a previously annotated intron (13). Alternative promoter and alternative terminator are two less commonly observed alternative splicing events in mammalian genomes (14). Transcripts from alternative promoter events have more than one initiator exon while alternative terminator event transcripts have more than one terminator exon (15). Despite the fact that more than nine categories of alternative splicing events have been described in the literature, typically only the five most common are utilized in the development of alternative splicing event detection algorithms such as MISO (16), SplAdder (17), and Bisbee (18).

While recent reviews have started to focus on alternative splicing events, few have centered heavily on how updated proteomics technologies can help detect alternative splicing events at the protein/proteoform level or how modern proteomic computational pipelines facilitate alternative splicing event detection (19). We believe that a thorough evaluation of the leading proteomics technologies will enable researchers to make informed decisions as they study alternative splicing events and their role in biology and disease. In this review, we first give an overview of the main sequencing technologies, RNA sequencing (RNA-seq) and mass spectrometry (MS)-based proteomics, emphasizing the performance of their short- and long-read approaches for alternative splicing event identification. We then elaborate on the advances in computational methods and tools designed for RNA-seq- and MS-based proteomics. For RNA-seq data analysis, we focus on splice variant detection, splicing outlier detection, and differential splicing analysis. As for MS-based proteomics, we discuss the construction of a customized protein database using RNA-seq data and the statistical approaches for alternative splicing event validation at the protein level. Last, we explore the unmet needs in splice variant detection and the challenges of current detection methods, and we suggest top-down (TD) MS proteomics as an emerging approach for alternative splicing event detection at the intact proteoform level.

## 2. TECHNOLOGIES FOR THE DETECTION OF SPLICE VARIANTS

**Figure 3:**
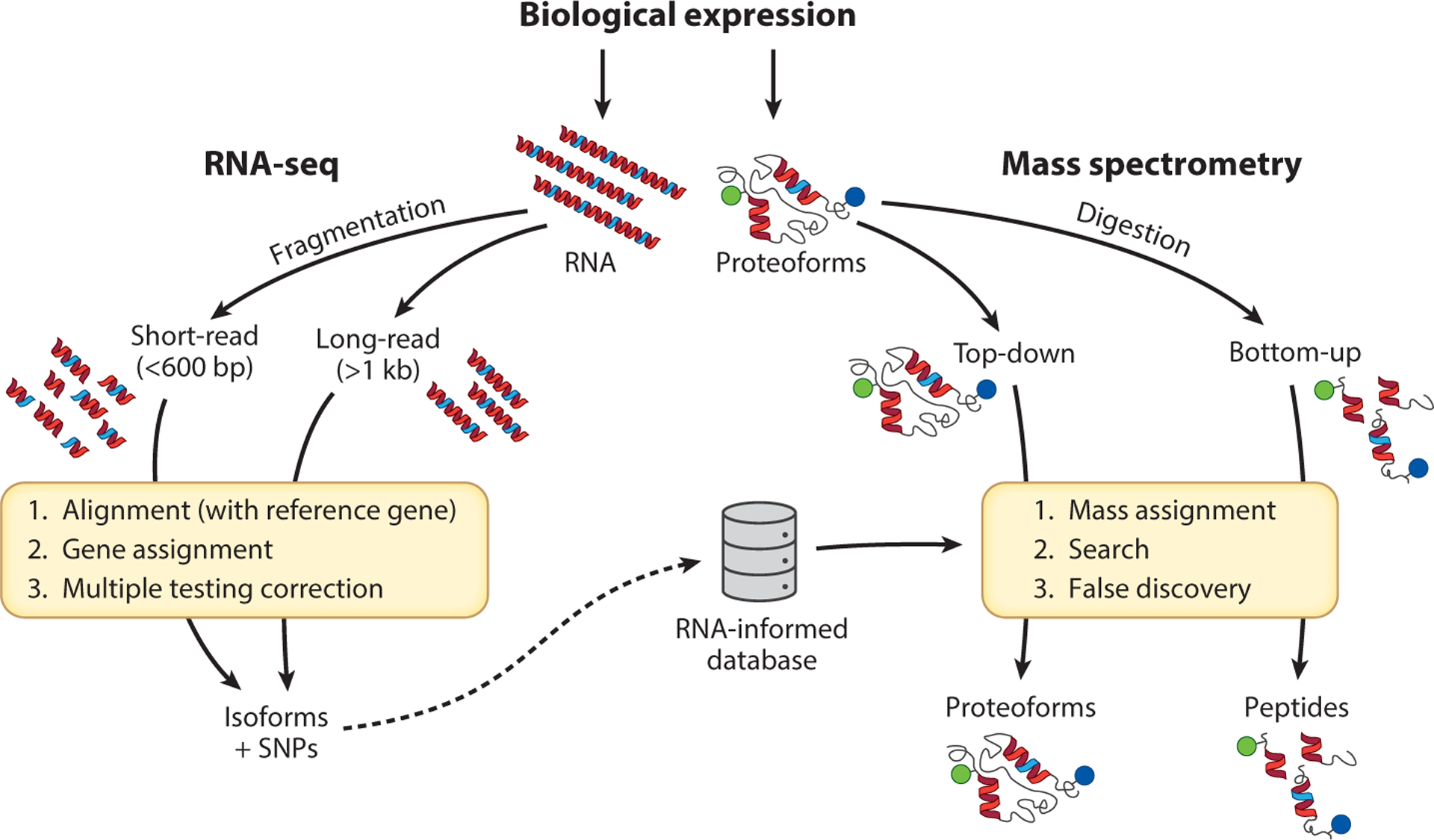
Short- and long-read sequencing approaches applied to RNA- and protein-level biology, which presents biomolecules for sequencing (*top*). Short-read sequencing came first for both RNA-seq (*left*) and so-called bottom-up proteomics based on peptides (*right*). Now, long-read options are available for asserting both alternative splicing in RNA (*left*) and protein isoforms (*right*) with higher confidence. Future synergies between these platforms for RNA-informed proteomics will be realized, as indicated by the dotted arrow (*bottom*). Abbreviations: bp, base pair; kb, kilobase pair; RNA-seq, RNA sequencing; SNP, single-nucleotide polymorphism.

While RNA-seq and MS-based proteomics are the two high-throughput ways to detect alternative splicing events, they work at different biological expression levels (Figure 3). RNA-seq is used to identify and quantify alternative splicing events at a transcriptomic level, while MS-based proteomics attempts to measure the products of alternative splicing, protein isoforms. Modern RNA sequencing generally can be categorized into short-read and long-read RNA-seq, with both methods reading complementary DNA (cDNA) sequences. However, short-read RNA-seq can only read cDNA fragments ranging between 75 and 600 base pairs (bp) in length, while long-read RNA-seq can read far longer cDNA with a maximum length of 30,000 bp, allowing many genes to be sequenced in their entirety.

An analogous dynamic exists in the MS field, between bottom-up (BU) and TD MS proteomics. In brief, in the widely used workflow of BU proteomics, which was developed in the Yates lab in the 1990s, proteins extracted from samples are digested by proteases such as trypsin or chymotrypsin into peptides and then injected into a mass spectrometer (20). In contrast, TD proteomics analyzes either denatured or native intact proteins without enzymatic digestion (21). Similar to long-read RNA sequencing, TD proteomics provides information on the full-length protein sequence, enabling a clear identification of protein isoforms and their individual proteoforms.

MS-based proteomics also serves as a technique to validate potential alternative splicing events previously identified by RNA-seq. Typically, the detection of MS-based alternative splicing products requires searching MS data against a customized protein database constructed based on existing RNA sequencing data (22, 23). These can be created from publicly available protein databases; however, these typically contain a limited number of protein isoform candidates and their PTMs. As the complexity of the search-space exponentially increases with the number of PTMs and their possible localizations, it is not usually possible to search for all possible PTMs. Therefore, a curated set of PTMs and other sources of protein variation is considered, especially for TD proteomics (24).

### 2.1. RNA Sequencing

Short-read RNA-seq typically utilizes next-generation sequencing (NGS) (25), one of the leading sequencing methods built upon a high-throughput sequencing platform first established by the Rothberg lab in 2005 (26), later commercialized by Illumina. Utilizing fiber-optic slides with more than a million picolitre-sized wells, this platform is capable of sequencing tens of millions of bases simultaneously (26). Since longer read lengths makes sequencing challenging, long-read RNA-seq requires third-generation sequencing technologies, initially developed by Pacific Biosciences around 2010 (25, 27). One key feature that differentiates third-generation sequencing from NGS is that third-generation sequencing does not require the amplification of DNA (27). Because short-read sequencing was developed earlier than long-read sequencing, many alternative splicing event detection algorithms and tools utilize short-read sequencing data as an input (28–31). Nevertheless, tools for analyzing long-read sequencing data have recently emerged, providing reads that cover the entire length of a transcript, enabling more accurate identification and quantification of alternative splicing events (32, 33). For example, minimap2 (32) and Magic-BLAST (33) are optimized for long-read sequencing data.

#### 2.1.1. Short-read RNA sequencing.

Since the development of short-read RNA sequencing in 2005 (34), many variants of RNA sequencing workflows have been developed; however, many of the core concepts have remained the same. In brief, RNA extracted from a sample is often used as a template in order to generate cDNA, as cDNA is more stable than RNA. This cDNA is then fragmented by physical methods (e.g., sonication) or enzymatic methods (e.g., nonspecific endonuclease), followed by size selection using 1% agarose gel to narrow the length to ~1 kilobase pair (kb) (35). Eventually, cDNA fragments are ligated to short oligonucleotides at both ends, facilitating polymerase chain reaction amplification before they are loaded on the sequencer. Sequenced cDNA fragments (typically known as reads) are then computationally assembled to infer full-length transcripts using either reference-based or de novo strategies. The reference-based method requires an existing genome or transcriptome database so the program can map reads to known sequences, while de novo assembly directly assembles reads to long transcripts by merging the reads with overlapping subsequences. Finally, a data matrix is generated, containing the detected transcripts and their reads, serving as an input for downstream analysis, including differential expression analysis and SNP calling, identifying genes with significantly different expression between experiment groups and identifying genes with SNPs, respectively (36, 37). Before the development of long-read RNA-seq technologies, the inability to sequence long stretches of DNA constrained the maximum fragment size of cDNA to between 75 bp and 600 bp (38).

Despite successfully detecting alternative splicing events at the RNA level in numerous biological and biomedical investigations, short-read RNA-seq is still limited due to the reliance on short reads during data analysis (39, 40). It has been demonstrated that assembling short reads into full-length transcripts is prone to errors, particularly when the RNA-seq data have poor sequencing coverage (39–41). Even if the sequencing depth and coverage are sufficiently high, short-read RNA-seq cannot determine the connectivity between exons when one gene has many isoforms because transcript assembly software can only infer, not directly detect, full-length isoforms at the protein level (40, 42, 43). Therefore, there remains a gap in our knowledge between what RNA-level isoforms are detected and what we actually know gets expressed into proteins (24).

#### 2.1.2. Long-read RNA sequencing.

In contrast to short-read RNA-seq, long-read RNA-seq can reach read lengths of up to 50 kb, although the maximum read length varies across long-read sequencing technologies (44). To date, two dominant independent long-read RNA sequencing technologies have been developed for real-time sequencing of DNA and RNA: Pacific Biosciences (PacBio) single-molecule real-time (SMRT) sequencing and Oxford Nanopore Technologies (ONT) (45, 46).

SMRT sequencing technology reads the sequence of cDNA based on real-time emitted light given off by fluorescently tagged nucleotides. Before reading, each of the four DNA nucleotides is tagged with one of four fluorescent dyes. When the nucleotides are assembled by a DNA polymerase using a cDNA fragment as a template, the fluorescent tag of each nucleotide is cleaved off, releasing one of four fluorescent signals, which are then recorded by the sequencer. SMRT has two modes, circular consensus sequencing (CCS) and continuous long read (CLR). In CCS, a circular single-strand cDNA molecule is formed by linking a double-strand cDNA at both ends mediated by two adapter sequences. Two primers will then anneal to the adapter sequences and start to transcribe the circularized DNA under the guide of a DNA polymerase. Since the cDNA is circular the transcription generates a long sequence containing multiple copies of the sequence of the cDNA. The sequence is then loaded to the reader after the adapter sequences are trimmed, resulting in multiple calls per base used to derive a high-quality read (47). It has been reported that CCS mode can sequence cDNA molecules whose lengths range from 10 kb to 20 kb with up to 99% read accuracy (46), although read accuracy is typically in the range of 75–90% (48). Alternatively, CLR sequencing mode allows the sequencing of the RNA greater than 20 kb (46). CLR also utilizes consensus sequence to increase the read accuracy but requires multiple copies of the same molecule in the sequencing pool and generates a single read for each molecule. This strategy enables the sequencing length of CLR mode to extend to 175 kb, although read accuracy is reduced to a maximum of 90%.

One feature distinguishing ONT sequencing from SMRT is that ONT devices are adapted to sequence RNA molecules directly, avoiding the errors and bias introduced during the reverse transcription and amplification. In the direct sequencing method, a poly(T) adapter, a short sequence that contains thymine only, is ligated to each extracted RNA strand’s poly(A) tail to start the reverse transcription. The transcribed cDNA and the original RNA form a cDNA-RNA duplex that stabilizes the RNA strand, followed by the direct ligation of the sequencing adapter at the 3´end of the RNA strand. The RNA strand will then pass through a nanopore made with a motor protein, a nanopore protein, and a membrane that holds the complex together. When an RNA molecule passes through this complex, an electrical signal will be generated and recorded, which can be analyzed to infer the RNA sequence. RNA direct sequencing is suitable for time-sensitive applications as it requires less sample manipulation (49). Moreover, direct sequencing is amplification-free and does not suffer from amplification errors and bias such as unequal amplifications and amplification artifacts. The sequencing length of ONT can reach 30 kb with reported raw read accuracies of greater than 99%, enabling highly accurate splice variant identification.

Although long-read RNA-seq technology captures many full-length transcripts, making this technique promising for splicing variant detection, several technological challenges remain unsolved. Both SMART and ONT nanopore sequencing suffer from lower read accuracy compared to short-read sequencing. Issues that lead to suboptimal read accuracy include but are not limited to an insufficient longevity of polymerase for long fragment transcription, a sequencing strategy that is vulnerable to sequence mutations, and the relative infancy of computational tools for error correction in data analysis (50).

#### 2.1.3. Alignment and mapping reads.

Alignment is a crucial step for alternative splicing event detection; this includes annotating RNA-seq reads with gene names, determining their relative abundance, and using the annotated reads as the input for downstream analysis. During the alignment process, reads are first subjected to quality control, removing reads that have low quality scores followed by mapping to a reference genome for transcript identification and counting (if the reference is available). At present, dozens of tools have been developed for RNA-seq read alignment (Table 1), many of which adopt similar strategies.

Typically, an alignment tool will initially index the genome, which involves splitting the whole reference genome into small fragments and saving the location and the exact sequence of each fragment in a data structure. The data structure utilized for storage has a large impact on alignment efficiency and memory usage, which has traditionally been a problem. Three common data structures used for genome indexing are the suffix tree (51), suffix array (52), and full-text index in minute space, also called FM-index (53), with FM-index using the least amount of memory to store genome information. FM-index utilizes the Burrow–Wheeler transformation to transform a fragment into a reversible and compressible string. This procedure allows for a human genome to be cached in a file with a size of less than 1 GB, dramatically reducing lookup times and RAM (random-access memory) requirements. Modern alignment tools such as Bowtie and Hisat2 have integrated FM-index into their workflows (54).

Along with the rapid development of long-read RNA-seq technology, many existing alignment tools, such as STAR, StringTie2, and Hisat2 have become compatible with processing long-read data. For instance, STAR has an extended version, STAR-long, in which read lengths can exceed 650 bp (the maximum length in traditional STAR). Nevertheless, people may encounter slow processing or interruption, possibly due to compatibility issues derived from different versions of programming languages or different formats of raw files. Several alignment tools such as Minimap2 and Magic-BLAST have been designed specifically for long-read data and are less prone to the issues encountered in short-read tools extended for long-read.

#### 2.1.4. Estimation of the percent splicing index.

The output of an alignment tool can inform users of the existence of alternative splicing products, but it does not yield information on the abundance of these products (55). In addition, the limitations of read length in short-read RNA-seq make the quantification of alternative splicing events difficult. However, by using the proper tools to measure alternative splicing events (Table 2), one can draw statistical conclusions about the relative abundance of different alternative isoforms in a sample. Comparative evaluations of these tools are available (56, 57); however, comparing performance benchmarks on reference datasets from different sources is often difficult, as standardization of parameters across software can be subjective.

A common way to identify and quantify alternative splicing events using short-read RNA-seq data is to calculate the percent splicing index (PSI or Ψ), which denotes the proportion of reads that represent the inclusion isoform, defined as the isoform that includes transcriptions of all the exons of one gene. In contrast, the exclusion isoform indicates that a given isoform arises from the skipping of one or more exons. In general, PSI calling algorithms take reads aligned to isoform sequences, obtained from reference genomes or through de novo assembly, and divides them into three categories: *N*_I_ and *N*_E_ correspond to the number of reads uniquely supporting the inclusion and exclusion isoform, respectively, and *N*_C_ corresponds to the number of reads supporting both isoforms.

In 2008, Wang et al. (58) described a way of estimating PSI (defined as Ψ_SJ_) which took into account reads mapped to alternative exons when calculating the density of reads supporting inclusion isoforms via read length normalization. To further improve the PSI estimation, Katz et al. (16) developed Ψ_MISO_, which considers constitutive reads, *N*_C_, during the ratio calculation. Previous PSI estimations only relied on reads unique to isoforms, but constitutive reads also contain latent information that can be used to stabilize PSI estimation. The MISO algorithm accounts for constitutive reads by recasting the analysis of isoforms as a Bayesian inference problem that estimates the proportion of the constitutive reads that belong to alternative isoforms, improving the measurement of exon expression.

#### 2.1.5. Estimation of the percent splicing index for complex splicing events.

Most PSI estimation methods are capable of quantifying the major types of alternative splicing events (Figure 2), but they are compromised when facing complex splicing events such as retained intron and multiple-exon-skipping events. In a retained intron event, a part of an intron is kept as part of an exon after the splicing event; thus, it is difficult to distinguish such reads from sequencing artifacts (59). Multiple-exon-skipping events are another complicated case, but these events are rare in comparison to single exon skipping. Nevertheless, these complex splicing events have been shown to cause severe diseases in humans (60, 61). Therefore, several modifications have been applied to the regular PSI estimation for the quantification of complex splicing events.

Intron-centric PSI estimation, Ψ_Intron-centric_, allows the detection and quantification of retained intron events and was first proposed by Pervouchine et al. (62). This idea has once again come under the spotlight due to short-read RNA-seq revealing that retained intron events are linked to many human diseases such as Alzheimer’s disease and prostate cancers (63, 64). Compared with other PSI estimations, intron-centric PSI is superior at detecting partial or full intron retention (64, 65). Since intron-centric PSI enables the detection of the majority of alternative splicing types, it has been integrated into other algorithms or workflows such as FRASER to detect aberrant splicing events in patients (66–68).

In 2019, Lin & Krainer (69) developed a free software tool, PSI-Sigma, for the detection of multiple exon-skipping events. Traditional PSI estimation only uses reads from splice junctions and the alternative exons derived from one specific isoform. In order to detect multiple exon-skipping events, PSI-Sigma considers the junction reads of all isoforms between two exons and is compatible with detecting other alternative splicing events such as single-exon-skipping and retained intron events.

#### 2.1.6. Differential splicing analysis.

Differential splicing analysis utilizes a calculated PSI value to infer the difference between an alternative splicing event within replicates and one between sample groups. The difference of two PSI values of the same gene, Δ*PSI*, across individual samples is usually used as an indicator in differential splicing analysis. To ascertain statistical significance in these differences, researchers have developed dedicated algorithms to perform differential splicing analysis (70, 71).

In 2012, Shen et al. (72) developed MATS (multivariate analysis of transcript splicing), a Bayesian statistical framework for flexible hypothesis testing of differential alternative splicing patterns on short-read RNA-seq data, allowing for the statistically rigorous analysis of the sequence expression changes between samples/treatment groups. Instead of optimizing the estimation of PSI, MATS determines whether the Δ*PSI* between two samples exceeds a user-defined threshold and calculates two metrics: a *p*-value (indicating the probability that the observation is the result of chance), and a subsequent false discovery rate (FDR) associated with the threshold. By performing rigorous statistical analysis, MATS minimizes ambiguity when determining whether calculated PSI values between two experimental conditions are significantly different from each other, typically referred to as hypothesis testing.

MATS calculates PSI, Ψ_MATS_, in a way similar to Ψ_SJ_. In a two-sample case, for each alternative splicing event, MATS calculates PSI Ψ_1_ and Ψ_2_ for the first and the second sample, respectively, and assigns a *p*-value to this ΔPSI using a Bayesian framework. As there are typically many of these hypothesis tests, a multiple-testing correction must be applied; MATS applies the Benjamini–Hochberg method (73) in order to generate a list of *Q*-values, thereby controlling the FDR. MATS has been further optimized to take into account biological replicates, as they can drastically influence PSI estimation (70). To model and correct the deviation within paired and unpaired replicates, the rMATS algorithm was developed and has fully replaced MATS as of 2011.

By applying PSI estimation and differential splicing analysis to RNA-seq data, users can confidently identify and quantify alternative splicing events in samples at a transcriptomic level. However, it is protein isoforms with PTMs that produce phenotypic responses. Without the identification of the products of alternative splicing events at the protein level, there will remain gaps between the transcriptome, proteome, and phenotypes in research. MS-based proteomics is the leading method to identify and quantify the proteins and proteoforms in organisms; thus, it is meaningful to discuss its application in alternative splicing events detection.

### 2.2. Mass Spectrometry

Unlike RNA-seq techniques, which read the sequence of cDNA, the mass spectrometer is an analytical instrument that measures the mass-to-charge (*m*/*z*) ratio of ionized molecules in a sample. MS data for protein analysis generally consist of two acquisitions, MS^1^ and MS^2^. In MS^1^ acquisition, the protein or peptide molecules are first ionized by electrospray to form a stream of ions that flow into the mass spectrometer (74). The injected ions within the user-defined range of *m*/*z* values will be selected and delivered to the mass analyzer. Ions measured in an MS^1^ scan are typically named precursor ions and represent the intact peptide or proteoform. To obtain MS^2^ spectra, one first isolates precursor ions by their *m*/*z* in the gas phase, and then their covalent bonds are fragmented and the *m/z* and intensity values of their product ions are recorded. Several strategies are available for precursor isolation such as inclusion list, data-dependent acquisition, and data-independent acquisition (75). Precursor ions are fragmented by a variety of fragmentation techniques using collisions with neutral gas or electron manipulation, such as collision-induced dissociation, higher-energy collisional dissociation, or electron transfer dissociation. The MS^1^ and related MS^2^ spectra are then deconvoluted into the mass domain to serve as input for database searching and downstream analyses.

#### 2.2.1. Bottom-up and top-down proteomics.

BU and TD proteomics, the two dominant MS techniques, take fundamentally different approaches to sample preparation: BU proteomics analyzes small peptides created by enzymatic digestion whereas TD proteomics analyzes the intact proteins directly (no digestion). Because it deals with small peptides, BU proteomics is considered a shotgun proteomics approach analogous to shotgun genome sequencing (76). The application of BU proteomics can be traced back to 2001 when the Yates lab successfully identified more than one thousand proteins in yeast (77). In BU proteomics, proteins extracted from a sample are usually subjected to denaturing, reduction, alkylation, digestion, and desalting (20). Before the MS analysis, the resulting peptide mixture is separated by a liquid chromatography (LC) system in order to decrease the sample complexity when the sample reaches the mass spectrometer (76). One big advantage of BU proteomics is that it can identify and quantify tens of thousands of short peptides in a single run (78); however, it typically provides only <10% sequence coverage of the whole human proteome and does not differentiate peptides that are shared by different isoforms and proteoforms (21). Therefore, unique isoforms cannot be inferred with clarity.

Compared to BU proteomics, TD proteomics is a rising technology that is beginning to advance biomedical studies (21, 79–82). For example, in 2021, the Ge lab managed to characterize metabolic enzymes in heart tissues using a TD proteomics platform coupled with serial size exclusion chromatography (83). Sample preparation in TD proteomics typically requires fewer steps than BU proteomics, reducing the number of experimental artifacts (84). The main factor that influences the selection of sample processing methods is the complexity of the sample itself, that is, the number of distinct proteoforms present. If the sample is largely homogenous, then only a simple cleaning to remove nonvolatile salt followed by dilution in an MS-compatible buffer is required. As the number of proteoforms climbs toward 100 (they usually are derived from a fraction of cell lysate), precipitation using organic solvent should be included to isolate proteins from other soluble small molecules (e.g., salts and detergents). If the sample contains hundreds of proteoforms or more, offline prefractionation may be used to decrease sample complexity. In TD proteomics, common fractionation approaches are polyacrylamide gel–based prefractionation [PEPPI-MS (passively eluting proteins from polyacrylamide gels as intact species for MS)] or size exclusion chromatography (83, 85). After preparation, the sample can be injected into the mass spectrometer using online separation techniques such as LC and capillary electrophoresis.

#### 2.2.2. Pros and cons in splice variants identification using mass spectrometry.

BU proteomics generally has three common caveats in detecting splice variants (81, 82). First, BU may not detect peptides that differentiate one isoform from another, as these peptides may be lost during sample preparation or are insensitive to MS analysis. Second, several related gene products may share the same peptide sequence, making the exact origin of the peptide unknown. Third, even if unique peptides are identified for multiple isoforms, their relative abundance remains unclear, as it is challenging to assign the correct proportion of any shared peptides to different protein isoforms (86). In an attempt to address this, PeptideClassifier was developed to decrease ambiguity when assigning peptides to proteins, but uncertainty remains given that not all shared peptides can be classified (87). In contrast, TD proteomics does not suffer from such limitations. Instead, TD proteomics detects intact protein molecules, leaving far less ambiguity about the origin of the fragments and the combination of PTMs. This lack of ambiguity can lead to the direct identification of proteoforms rather than peptides.

Nevertheless, TD proteomics does have some shortcomings when compared with BU proteomics. In TD, the researcher usually yields thousands of proteoform identifications (IDs) from hundreds of proteins with proper fractionation, while BU proteomics can readily provide around 4,000–8,000 inferred protein IDs based on 30,000–100,000 short peptides. In addition, TD proteomics does not perform well on identifying proteins with molecular weight >40 kDa in complex mixtures due to the fast-decaying signal-to-noise ratio with increasing molecular weight (88). Nevertheless, recent developments have extended this range to around 200 kDa by using a serial size exclusion chromatography strategy (89, 90).

#### 2.2.3. Database construction.

Protein or proteoform database construction is one of the key steps that determine whether MS raw data can be converted into intuitive protein or proteoform identifications, especially in the case of alternative splicing event identification. A protein database usually contains simple protein sequences; however, it is possible to construct a proteoform database that contains PTM and SNP information associated with protein sequences. If the database only contains sequences of canonical proteins, researchers will be unable to see many potential targets of interest. In contrast, if the database contains all possible alternative splicing species for every protein, the database search space can quickly expand beyond a practical limit, especially when one considers all possible PTM combinations. One simple way to make a database for searching is to download a proteome of interest from publicly available knowledgebases such as UniProtKB and PhosphoSitePlus (91, 92). UniProtKB consists of two separate repositories: TrEMBL, containing computationally generated sequences, and SwissProt, containing manually curated sequences. These resources offer numerous protein sequences including canonical sequences and common alternative splicing species for each protein in popular formats such as FASTA (protein sequences only) and XML (sequences, PTMs, and SNPs) (91). A method of constructing sample specific databases was first proposed by Wang et al. (93) in 2012, in which they first performed short-read RNA-seq in patient samples and translated all identified transcripts into protein sequences for protein database construction (Figure 3, *dotted line*). This workflow has quickly been applied to many biological and biomedical studies given its advantages over traditional methods in identifying unknown and novel proteoforms (94). In addition, RNA-seq data also provide information on SNPs in a sample. SNPs are highly patient specific and highly variable; therefore, it is impossible to include all possible SNPs in a traditional database. By using a multi-omics approach, researchers can accurately and efficiently identify mutated proteins in patients (95). Along with the developments of RNA-seq and MS technologies, other types of NGS techniques such as single-cell RNA-seq and long-read RNA-seq have also been integrated with MS techniques (96, 97).

An alternative way to identify protein isoforms is de novo sequencing, in which the protein sequence is generated directly from the MS spectra without searching against a database. De novo sequencing performs best when users have samples with low complexity and high sequence coverage. For example, Vyatkina et al. (98) observed 90% sequencing coverage for carbonic anhydrase and the F_ab_ region of alemtuzumab, an antibody used to treat chronic lymphocytic leukemia. Since contemporary ion dissociation approaches used in TD proteomics rarely produce fragmentation patterns with >90% sequence coverage, de novo sequencing in TD proteomics is still developing (99). To overcome the lack of high sequence coverage, Liu et al. (100) developed the TBNovo software package, enabling de novo sequencing by combining TD and BU data. TBNovo uses TD proteomics data to define a backbone of an intact protein with limited sequence coverage and aligns each BU-identified peptide to this backbone to increase the sequence coverage. Another de novo sequencing method developed by Vyatkina et al. applies the concept of sequence tag convolution to generate a long but reasonably gapped protein sequence (101, 102). Nevertheless, de novo sequencing utilizing TD proteomics data remains underdeveloped.

#### 2.2.4. Database searching.

Database searching in BU proteomics takes MS spectral data and a database of protein sequences as input and generates peptide identifications from which proteins may be inferred (76). In TD proteomics, spectral data from fragmented protein ions are typically searched against a database of candidate proteoforms that contain isoform sequences, SNPs, and PTMs, yielding a list of proteoforms IDs containing protein isoform sequences and localization information of their PTMs and SNPs. For both proteomic approaches, IDs are typically ranked using a scoring metric indicating the confidence of each identification.

As TD proteomics has started to grow in popularity, some BU proteomics analysis tools have developed additional modes in order to be compatible with TD proteomics data (Table 3). For example, in recent years the Mascot search engine, originally designed for searching BU data, has extended the precursor mass limit from 16 kDa to 110 kDa, increasing compatibility with TD proteomics data without affecting the searching performance for BU experiments (103, 104). Other tools like SEQUEST and OMSSA are also somewhat compatible with TD proteomics data, but they are not commonly used for TD analysis (105–107).

A handful of database-searching programs have been developed specifically for proteoform identification using TD proteomics data (Table 3). For example, MS-Align+ utilizes a spectral alignment approach that aligns a given protein sequence against all observed spectra (106, 108). The algorithm then tries to find the highest-scoring alignment between the sequence and the spectrum. One advantage of this method is that researchers can add additional PTMs that are not included in the database, facilitating PTM discovery, which is challenging in proteomics generally. Another TD-specific proteomic suite, TopPIC, utilizes a filtering algorithm based on a linked list data structure and an alignment algorithm adapted from the one utilized in MS-Align+ with additional database indexing to increase speed (106, 108, 109). One of the first spectral alignment algorithms, SEQUEST, utilizes cross-correlation in order to score spectral matches (XCorr) (110). This XCorr approach allows the use of low-resolution spectral data, opening proteomics to a wider variety of MS instruments, albeit at a cost of significantly longer processing times (111).

Another commonly used database searching program for TD spectral analysis is ProSightPD. This application offers three types of searches: absolute mass, biomarker, and sequence tag. The most used search mode is absolute mass, which first locates a proteoform whose theoretical mass matches the observed precursor ion’s mass and then compares all theoretical fragments of each located proteoform with those derived from the observed precursor ions. The biomarker search looks for the observed MS spectra that match subsequences/peptides of any proteoforms in the database. The major reason to implement a biomarker search is to detect proteolytic polypeptides (e.g., amyloid beta peptide in Alzheimer’s disease) and truncated proteins in samples (112–114). Another search mode, sequence tag search, first reported in 1996 by McLafferty, Mann, and colleagues (115), extrapolates a peptide sequence from a sequential fragmentation and searches the predicted peptide against a database. Although the sequence tag search enables the generation of peptide sequences without doing database search, the algorithm deeply relies on high-quality fragmentation with great sequence coverage.

#### 2.2.5. Statistical analysis of search results: automated and confident proteoform assignment.

Confidence in proteoform identification is vital for researchers to be able to draw relevant biological conclusions, such as the detection of alternative splicing events and isoform-specific modifications. However, automated searching algorithms are prone to false positives and ambiguity, especially with automatically generated data with high variance in its quality. Therefore, database search tools need robust statistical analysis associated with these IDs and PTM localizations to support any biological interpretations.

To estimate the probability of obtaining at least as good a match as chance between the observed and theoretical fragments, Meng et al. developed the P-score (116), which has been implemented in ProsightPD (117). Utilizing a Poisson distribution, the P-score estimates the specificity of a match returned by the search engine, with a lower P-score indicating a better match, as the probability to have this many fragments randomly matched to a sequence is low.

A typical proteomic experiment will result in hundreds or even thousands of matches, each with individual scoring metrics demonstrating the statistical probability of seeing that one match by random chance, usually known as a hypothesis testing. In a scenario where one is testing multiple hypotheses at the same time the likelihood of a false positive will increase. This is known as the multiple testing problem and requires a correction to control the false positive rate.

The FDR is another critical statistic to evaluate search results that gives the expected proportion of false positives in the returned list of tested hypotheses (118). One controlling procedure for FDR is the Bonferroni correction, which is utilized in E-value calculations (119) and is simply the P-score multiplied by the total number of proteoforms in the search space; however, this is conservative, as it removes more true positives than other methods. A less conservative but more appropriate approach is the Benjamini–Hochberg (BH) procedure, which is a sequential Bonferroni correction (73). Compared to the Bonferroni correction, the BH procedure considers the rank of *p*-values of all entries and modifies the Bonferroni correction by rank. This correction performs well in both MS and RNA-seq analyses, especially when users are performing differential expression or multiple comparison analyses, allowing for greater confidence in both isoform and proteoform identification. In 2019, LeDuc et al. developed a context-dependent FDR calculator (120) that estimates FDR at four molecular levels of study: proteoform spectral match, protein, isoform, and proteoform in TD proteomics. This research suggested that the isoform-level FDR should be calculated when researchers use TD proteomics for splice variant detection.

## 3. CONCLUSIONS AND OUTLOOK

Confidently detecting alternative splicing events in biological samples using omics approaches has been a major challenge for more than 20 years. At the transcriptomic level, the evolution of PSI estimation opened new opportunities for biologists to investigate much more complex alternative splicing events using short-read RNA-seq. The newly developed long-read RNA-seq technologies detect alternative splicing events more accurately and with greater confidence than short-read RNA-seq, enabling unambiguous identification of unannotated transcripts and alternative splicing in long transcripts. While genomic information reveals the crucial roles of genes in different biological contexts, it is proteoforms that report more faithfully on molecular information underlying complex human diseases. Therefore, it is important to discuss the advances in mass spectrometry techniques that have elevated the detection of alternative splicing events to an isoform/proteoform level. Just like the dynamics between short-read and long-read RNA-seq, the measurement of intact proteins using TD proteomics does not suffer from the loss of peptides and ambiguity during the sequence alignment, making the detection of isoforms far more robust. Proteogenomics is a growing study strategy integrating NGS techniques with MS-based proteomics (121) and has increased the accuracy of alternative splicing event detection in biological samples by making sample-specific protein/proteoform databases for MS spectral searching.

Major changes in sequencing and computational methods analyzing transcriptomic and proteomic data have resulted in the development of hundreds of data analysis tools (84, 122). New tools are required in order to capture and integrate new data types, particularly for two emerging technologies, long-read RNA-seq and TD proteoform analysis. Their combination offers unique opportunities to study complex alternative splicing events, but challenges remain in both technologies. To alleviate limitations, researchers can focus on building platforms where popular data analysis tools will be better connected and interoperable to extend the availability of one-stop workflows (123). Large-scale funding in this area based on a consortium model may enable the emergence of a clarified solution in this space (124, 125).

Recent improvements of RNA-seq and protein-sequencing technologies are helping to better understand proteoform and isoform expression in the human genome/proteome, and this area will be particularly assisted by mapping reads and proteoforms to the Telomere-to-Telomere consortium’s gapless human genome, which was completed in 2022 for both euchromatic and heterochromatic regions (126). In addition to human genome information, the mapping of the human proteoform landscape is of great importance and a clear next step after the genome (127). With the accelerating development of TD-MS technologies, the Human Proteoform Atlas (128) and the Blood Proteoform Atlas (80) were launched recently. These projects indicate that proteoforms better describe protein-level biology than do their corresponding proteins. By reviewing the state-of-the-art transcriptomics and proteomics technologies, we hope to demonstrate that there is a great opportunity to assert human isoforms and proteoforms with complete molecular specificity.

## Figures and Tables

**Table 1:** Software applications for alignment and read mapping of short-read sequences to assert alternative RNA transcripts

Software	Language	Alignment algorithm	SR/LR	Indexing	Publication date	Last update	Notes	Reference(s)
STAR	C, C++	BWT	+/+	Suffix array	January 2013	January 2022	LR version available: STAR-long	23
Hisat2	Python	BWT	+/−	Graph F-index	August 2019	July 2020	Successor to HISAT and TopHat2	25
Minimap2	C, Python	BWT	+/+	Hashing	May 2018	Dec 2021	Maps DNA-seq reads against a large reference database	27
TopHat2	C++	BWT	+/−	FM-index	April 2012	Feb 2016	Improved version of TopHat	24
Magic-BLAST	C++	BWT	+/+	Hashing	July 2019	May 2021	Maps DNA-seq reads against a whole genome	28
Bowtie2	C++	BWT	+/+	FM-index	March 2009	January 2022	Successor to Bowtie	54, 129
BWA-mem2	C++	BWT	+/+	FM-index	May 2019	March 2021	A plugin for BWA aligner	130
Subread	C	BWT	+/+	Hashing	May 2013	July 2021	Aligns DNA- and RNA-seq reads; identifies genomic mutations including short indels and structural variants	131
Salmon	C++	BWT	+/−	Suffix array	Sep 2017	Jun 2022	Uses a reference genome to make a decoy database for the alignment	132
BBMAP	Java	SW	+/+	Hashing	Feb 2014	Oct 2022	Splice-aware global aligner for DNA- and RNA-seq reads	133
GMAP	C	Oligomer chaining	+/+	Hashing	March 2005	Dec 2021	Supports the long-read alignment. In 2016, the author also developed another tool, GSNAP, for short-read alignment	134, 135

Abbreviations: BWT, Burrow–Wheeler transformation; LR, long-read; -seq, sequencing; SR, short-read; SW, Smith–Waterman.

**Table 2:** Software applications developed to process short- or long-read RNA-seq data types into mapped reads and alternative splicing calls

Software	Language	PSI estimation method	Replicates supported	Publication date	Last update	Reference
MISO	Python	Ψ_MISO_	−	Nov 2010	July 2017	16
rMATS	Python	Ψ_MATS_	+	Dec 2011	Dec 2021	70
PSI-Sigma	Perl, Raku	Ψ_Sigma_	+	Dec 2019	Aug 2022	69
FRASER	R	Ψ_Intron-centric_	+	Jan 2021	Jun 2022	66
SUPPA2	Python	Ψ_SJ_	+	Mar 2018	Feb 2018	71
MAJIQ	Python	Ψ_SJ_	+	Feb 2016	Nov 2019	136
Whippet	Julia	Ψ_SJ_	+	Oct 2018	Mar 2021	137

Abbreviation: PSI, percent splicing index; RNA-seq, RNA-sequencing.

**Table 3:** Software tools to convert short- and long-read proteomics data (commonly called bottom-up and top-down proteomics) into information on protein isoforms and whole proteoforms

Software	Language	BU/TD	Required data type	Database input type	Publication date	Last update	Reference
Mascot	C	BU/TD	Thermo RAW	FASTA	2008	2022	103
ProSightPD	C#	TD	Thermo RAW	FASTA, XML	2004	2022	^a^
TopPIC	C++	TD	mzML	FASTA	2016	2022	108
pTop	C++	TD	Thermo RAW, MGF	FASTA	2016	2016	138
TopMG	C++	TD	mzML	FASTA	2017	2022	139
MSPathFinder	C#	TD	Thermo RAW(more with ProteoWizard)	FASTA	2017	2022	140
MetaMorpheus	C#	TD	Thermo RAW, mzML	FASTA, XML	2018	2021	141

a https://www.proteinaceous.net/prosightpd.

Abbreviations: BU, bottom-up; TD, top-down.
